# Dimethyl 2-(3-chloro­phen­yl)-6-hy­droxy-6-methyl-4-(methyl­amino)­cyclo­hex-3-ene-1,3-dicarboxyl­ate

**DOI:** 10.1107/S1600536811017089

**Published:** 2011-05-14

**Authors:** S. Amirthaganesan, S. Sundaramoorthy, D. Velmurugan, Y. T. Jeong

**Affiliations:** a Department of Image Science and Engineering, Pukyong National University, Busan, 608-739, Republic of Korea; b Centre of Advanced Study in Crystallography and Biophysics, University of Madras, Guindy Campus, Chennai 600 025, India

## Abstract

In the title compound, C_18_H_22_ClNO_5_, the cyclo­hexene ring adopts a distorted half-chair conformation. The mol­ecular structure is stabilized by pairs of intra­molecular N—H⋯O and O—H⋯O inter­actions, generating *S*(6) motifs. In the crystal, the mol­ecules are linked by inter­molecular C—H⋯O inter­actions, forming centrosymmetric dimers.

## Related literature

For the synthesis see: Pandiarajan *et al.* (2005 ▶). For related structures, see: Amézquita-Valencia *et al.* (2009, ▶ 2010) ▶; Venter *et al.* (2010 ▶). For ring conformational analysis, see: Cremer & Pople (1975 ▶); Nardelli (1983 ▶). For hydrogen-bond motifs, see: Bernstein *et al.* (1995 ▶).
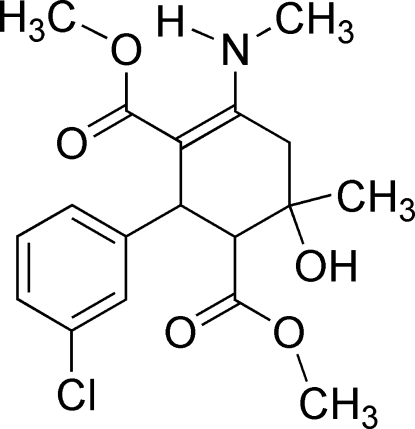


## Experimental

### 

#### Crystal data

C_18_H_22_ClNO_5_
*M* *_r_* = 367.82
Monoclinic, 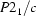

*a* = 11.962 (3) Å
*b* = 9.118 (4) Å
*c* = 17.704 (5) Å
β = 104.890 (3)°
*V* = 1866.1 (11) Å^3^
*Z* = 4
Mo *K*α radiation
μ = 0.23 mm^−1^
*T* = 293 K
0.25 × 0.22 × 0.2 mm

#### Data collection

Bruker SMART APEXII area-detector diffractometer
Absorption correction: multi-scan (*SADABS*; Bruker, 2008 ▶) *T*_min_ = 0.944, *T*_max_ = 0.955
17431 measured reflections
4638 independent reflections
3337 reflections with *I* > 2σ(*I*)
*R*_int_ = 0.024

#### Refinement

*R*[*F* ^2^ > 2σ(*F* ^2^)] = 0.044
*wR*(*F* ^2^) = 0.138
*S* = 1.04
4638 reflections
231 parameters
H-atom parameters constrained
Δρ_max_ = 0.35 e Å^−3^
Δρ_min_ = −0.46 e Å^−3^

### 

Data collection: *APEX2* (Bruker, 2008 ▶); cell refinement: *SAINT* (Bruker, 2008 ▶); data reduction: *SAINT*; program(s) used to solve structure: *SHELXS97* (Sheldrick, 2008 ▶); program(s) used to refine structure: *SHELXL97* (Sheldrick, 2008 ▶); molecular graphics: *ORTEP-3* (Farrugia, 1997 ▶); software used to prepare material for publication: *SHELXL97* and *PLATON* (Spek, 2009 ▶).

## Supplementary Material

Crystal structure: contains datablocks global, I. DOI: 10.1107/S1600536811017089/bt5540sup1.cif

Structure factors: contains datablocks I. DOI: 10.1107/S1600536811017089/bt5540Isup2.hkl

Additional supplementary materials:  crystallographic information; 3D view; checkCIF report

## Figures and Tables

**Table 1:** Hydrogen-bond geometry (Å, °)

*D*—H⋯*A*	*D*—H	H⋯*A*	*D*⋯*A*	*D*—H⋯*A*
N1—H1*A*⋯O1	0.86	2.0	2.673 (2)	134
O3—H4*A*⋯O4	0.82	2.39	2.990 (2)	131
C15—H15*B*⋯O1^i^	0.96	2.52	3.327 (3)	142

Symmetry code: (i) 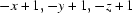.

**Fig. 1.**
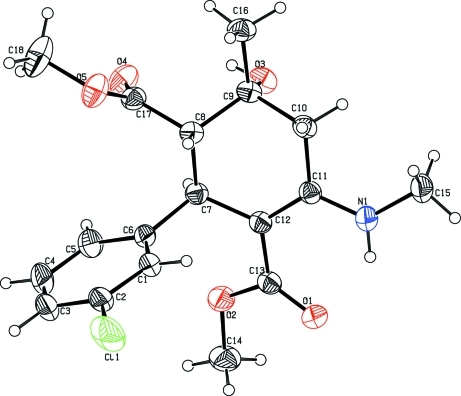
Perspective view of the molecule showing the thermal ellipsoids are drawn at 30% probability level.

**Fig. 2.**
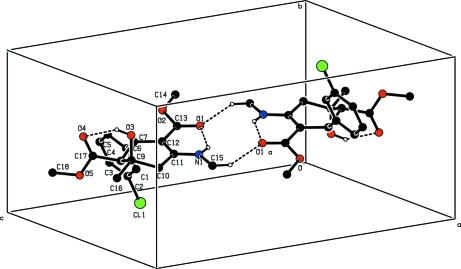
The crystal packing of the molecules viewed along a face. For clarity, hydrogen atoms which are not involved in hydrogen bonding are omitted

## supplementary crystallographic information

### Comment

The title compound has been obtained as a minor product during the synthesis of 2,4-bismethoxycarbonyl-3-(3-chlorophenyl)- 5-hydroxy-5-methylcyclohexanone (Pandiarajan *et al.*, 2005). The synthesized cyclohexanone has been purified by the recrystallization process in ethanol solvent. The expected compound regenerated and settled as powder along with some small crystals. The obtained crystals were analysed by single-crystal XRD and the results clearly evidence the formation of the title compound.

The *ORTEP* diagram of the title compound is shown in Fig.1. The cyclohexane ring adopts a distorted half-chair conformation with the puckering parameters (Cremer & Pople, 1975) and the smallest displacement asymmetry parameters (Nardelli, 1983) being q_2_ = 0.4038 (16) Å, q_3_ = -0.3083 (16) Å, Q_T_ = 0.5080 (16) Å, and θ = 127.36 (18)°. Atom Cl1 deviates from the plane of the C1—C6 benzene ring by 0.034 (1) Å.

The crystal packing is stabilized by C—H···O intermolecular interactions. The molecular structure is stabilized by N—H···O and O—H···O hydrogen bonds, wherein, atom N1 and O3 act as donor to O1 and O4, to generate *S*(6) motifs, respectively. In the crystal structure, the molecules at (*x*, *y*, *z*) and (1 - *x*,1 - *y*,1 - *z*) are linked by C(15)—H(15B) ···O(1) hydrogen bonds, generating a centrosymmetric dimeric ring motif *R_2_^2^*(14) (Bernstein *et al.*, 1995).

### Experimental

A mixture of 3-chlorobenzaldehyde (1 equvi) and methyl acetoacetate (2 equvi) and 40% methylamine solution (1 equvi) in ethanol was kept in hot water bath for about 30 minutes. It was kept aside for a day. The separated solid was recrystallized from ethanol.

### Refinement

The C bound H atoms positioned geometrically (C—H = 0.93–0.98 Å) and allowed to ride on their parent atoms, with 1.5*U*_eq_(C) for methyl H and 1.2 *U*_eq_(C) for other H atoms.

### Crystal data

**Table:**

C_18_H_22_ClNO_5_	*F*(000) = 776
*M**_r_* = 367.82	*D*_x_ = 1.309 Mg m^−^^3^
Monoclinic, *P*2_1_/*c*	Mo *K*α radiation, λ = 0.71073 Å
Hall symbol: -P 2ybc	Cell parameters from 1222 reflections
*a* = 11.962 (3) Å	θ = 1.8–28.3°
*b* = 9.118 (4) Å	µ = 0.23 mm^−^^1^
*c* = 17.704 (5) Å	*T* = 293 K
β = 104.890 (3)°	Block, colourless
*V* = 1866.1 (11) Å^3^	0.25 × 0.22 × 0.2 mm
*Z* = 4	

### Data collection

**Table:**

Bruker SMART APEXII area-detector diffractometer	4638 independent reflections
Radiation source: fine-focus sealed tube	3337 reflections with *I* > 2σ(*I*)
graphite	*R*_int_ = 0.024
ω and φ scans	θ_max_ = 28.3°, θ_min_ = 1.8°
Absorption correction: multi-scan (*SADABS*; Bruker, 2008)	*h* = −15→15
*T*_min_ = 0.944, *T*_max_ = 0.955	*k* = −7→12
17431 measured reflections	*l* = −23→23

### Refinement

**Table:**

Refinement on *F*^2^	Primary atom site location: structure-invariant direct methods
Least-squares matrix: full	Secondary atom site location: difference Fourier map
*R*[*F*^2^ > 2σ(*F*^2^)] = 0.044	Hydrogen site location: inferred from neighbouring sites
*wR*(*F*^2^) = 0.138	H-atom parameters constrained
*S* = 1.04	*w* = 1/[σ^2^(*F*_o_^2^) + (0.0627*P*)^2^ + 0.4709*P*] where *P* = (*F*_o_^2^ + 2*F*_c_^2^)/3
4638 reflections	(Δ/σ)_max_ = 0.009
231 parameters	Δρ_max_ = 0.35 e Å^−^^3^
0 restraints	Δρ_min_ = −0.46 e Å^−^^3^

### Special details

**Table:**

Geometry. All e.s.d.'s (except the e.s.d. in the dihedral angle between two l.s. planes) are estimated using the full covariance matrix. The cell e.s.d.'s are taken into account individually in the estimation of e.s.d.'s in distances, angles and torsion angles; correlations between e.s.d.'s in cell parameters are only used when they are defined by crystal symmetry. An approximate (isotropic) treatment of cell e.s.d.'s is used for estimating e.s.d.'s involving l.s. planes.
Refinement. Refinement of *F*^2^ against ALL reflections. The weighted *R*-factor *wR* and goodness of fit *S* are based on *F*^2^, conventional *R*-factors *R* are based on *F*, with *F* set to zero for negative *F*^2^. The threshold expression of *F*^2^ > σ(*F*^2^) is used only for calculating *R*-factors(gt) *etc*. and is not relevant to the choice of reflections for refinement. *R*-factors based on *F*^2^ are statistically about twice as large as those based on *F*, and *R*- factors based on ALL data will be even larger.

### Fractional atomic coordinates and isotropic or equivalent isotropic displacement parameters (Å^2^)

**Table:**

	*x*	*y*	*z*	*U*_iso_*/*U*_eq_	
C1	0.20024 (13)	0.2336 (2)	0.66810 (9)	0.0491 (4)	
H1	0.2693	0.1975	0.6606	0.059*	
C2	0.10067 (15)	0.1499 (2)	0.64705 (10)	0.0606 (5)	
C3	−0.00304 (16)	0.2004 (3)	0.65606 (12)	0.0762 (7)	
H3	−0.0696	0.1434	0.6412	0.091*	
C4	−0.00607 (16)	0.3366 (4)	0.68751 (14)	0.0845 (8)	
H4	−0.0757	0.3722	0.6944	0.101*	
C5	0.09225 (15)	0.4229 (3)	0.70946 (12)	0.0671 (5)	
H5	0.0881	0.5157	0.7303	0.081*	
C6	0.19700 (12)	0.37074 (19)	0.70030 (9)	0.0454 (4)	
C7	0.30800 (12)	0.45902 (17)	0.72953 (8)	0.0404 (3)	
H7	0.2868	0.5589	0.7408	0.048*	
C8	0.37441 (12)	0.38775 (16)	0.80710 (8)	0.0405 (3)	
H8	0.3766	0.2816	0.7989	0.049*	
C9	0.49940 (13)	0.44328 (17)	0.83388 (9)	0.0421 (3)	
C10	0.56091 (13)	0.39960 (18)	0.77252 (9)	0.0450 (3)	
H10A	0.6358	0.4475	0.7843	0.054*	
H10B	0.5743	0.2946	0.7758	0.054*	
C11	0.49636 (13)	0.43744 (17)	0.68999 (9)	0.0410 (3)	
C12	0.38012 (13)	0.46821 (16)	0.67106 (8)	0.0404 (3)	
C13	0.32399 (14)	0.51982 (17)	0.59341 (9)	0.0444 (3)	
C14	0.15531 (18)	0.6312 (3)	0.51469 (11)	0.0699 (5)	
H14A	0.1977	0.7082	0.4976	0.105*	
H14B	0.0818	0.6681	0.5184	0.105*	
H14C	0.1435	0.5519	0.4778	0.105*	
C15	0.68410 (14)	0.4189 (2)	0.65474 (11)	0.0597 (5)	
H15A	0.7225	0.4980	0.6869	0.090*	
H15B	0.7075	0.4172	0.6068	0.090*	
H15C	0.7045	0.3276	0.6818	0.090*	
C16	0.56123 (15)	0.3786 (2)	0.91304 (9)	0.0558 (4)	
H16A	0.6418	0.4041	0.9251	0.084*	
H16B	0.5533	0.2738	0.9112	0.084*	
H16C	0.5276	0.4172	0.9526	0.084*	
C17	0.31063 (14)	0.41559 (19)	0.86895 (9)	0.0469 (4)	
C18	0.2187 (3)	0.3077 (3)	0.95785 (16)	0.0975 (9)	
H18A	0.2735	0.3321	1.0061	0.146*	
H18B	0.1814	0.2168	0.9638	0.146*	
H18C	0.1618	0.3840	0.9442	0.146*	
N1	0.56045 (12)	0.43967 (18)	0.63774 (8)	0.0528 (4)	
H1A	0.5244	0.4549	0.5897	0.063*	
O1	0.36279 (10)	0.51675 (14)	0.53585 (7)	0.0560 (3)	
O2	0.21941 (10)	0.57946 (15)	0.58975 (7)	0.0584 (3)	
O3	0.50425 (11)	0.59997 (12)	0.83719 (7)	0.0542 (3)	
H4A	0.4653	0.6298	0.8660	0.081*	
O4	0.29242 (13)	0.53525 (15)	0.89139 (8)	0.0645 (4)	
O5	0.27827 (13)	0.29272 (15)	0.89645 (8)	0.0682 (4)	
Cl1	0.10795 (6)	−0.02490 (8)	0.60884 (5)	0.1010 (3)	

### Atomic displacement parameters (Å^2^)

**Table:**

	*U*^11^	*U*^22^	*U*^33^	*U*^12^	*U*^13^	*U*^23^
C1	0.0376 (8)	0.0622 (10)	0.0469 (8)	−0.0061 (7)	0.0098 (6)	0.0010 (8)
C2	0.0497 (9)	0.0767 (13)	0.0517 (10)	−0.0198 (9)	0.0062 (8)	0.0058 (9)
C3	0.0414 (10)	0.119 (2)	0.0647 (12)	−0.0225 (11)	0.0068 (8)	0.0159 (13)
C4	0.0342 (9)	0.140 (2)	0.0821 (15)	0.0098 (12)	0.0193 (9)	0.0128 (16)
C5	0.0430 (9)	0.0944 (15)	0.0655 (11)	0.0141 (10)	0.0169 (8)	−0.0020 (11)
C6	0.0350 (7)	0.0616 (10)	0.0400 (7)	0.0044 (7)	0.0102 (6)	0.0052 (7)
C7	0.0387 (7)	0.0420 (8)	0.0394 (7)	0.0054 (6)	0.0081 (6)	−0.0008 (6)
C8	0.0421 (7)	0.0376 (7)	0.0403 (7)	0.0038 (6)	0.0079 (6)	−0.0009 (6)
C9	0.0426 (8)	0.0385 (8)	0.0417 (7)	0.0042 (6)	0.0044 (6)	−0.0030 (6)
C10	0.0374 (7)	0.0481 (8)	0.0466 (8)	0.0037 (6)	0.0055 (6)	−0.0020 (7)
C11	0.0403 (7)	0.0386 (7)	0.0437 (8)	−0.0035 (6)	0.0102 (6)	−0.0024 (6)
C12	0.0397 (7)	0.0400 (7)	0.0403 (7)	−0.0017 (6)	0.0082 (6)	−0.0007 (6)
C13	0.0426 (8)	0.0434 (8)	0.0448 (8)	−0.0040 (6)	0.0067 (6)	−0.0010 (6)
C14	0.0613 (11)	0.0841 (14)	0.0560 (10)	0.0188 (10)	−0.0002 (9)	0.0116 (10)
C15	0.0439 (9)	0.0733 (12)	0.0662 (11)	0.0014 (8)	0.0220 (8)	0.0039 (9)
C16	0.0540 (9)	0.0634 (11)	0.0444 (8)	0.0121 (8)	0.0024 (7)	0.0013 (8)
C17	0.0467 (8)	0.0512 (9)	0.0415 (8)	0.0065 (7)	0.0089 (6)	0.0045 (7)
C18	0.119 (2)	0.0965 (19)	0.1025 (18)	0.0165 (16)	0.0740 (17)	0.0306 (15)
N1	0.0410 (7)	0.0695 (9)	0.0493 (8)	−0.0001 (6)	0.0142 (6)	0.0047 (7)
O1	0.0550 (7)	0.0707 (8)	0.0418 (6)	−0.0006 (6)	0.0115 (5)	0.0031 (6)
O2	0.0510 (7)	0.0724 (8)	0.0494 (6)	0.0148 (6)	0.0085 (5)	0.0126 (6)
O3	0.0582 (7)	0.0396 (6)	0.0613 (7)	0.0005 (5)	0.0093 (6)	−0.0078 (5)
O4	0.0821 (9)	0.0578 (8)	0.0607 (8)	0.0108 (7)	0.0313 (7)	−0.0045 (6)
O5	0.0826 (9)	0.0585 (8)	0.0754 (9)	0.0083 (7)	0.0420 (7)	0.0149 (7)
Cl1	0.0875 (4)	0.0874 (5)	0.1206 (5)	−0.0422 (3)	0.0133 (4)	−0.0251 (4)

### Geometric parameters (Å, °)

**Table:**

C1—C6	1.379 (2)	C11—N1	1.345 (2)
C1—C2	1.383 (2)	C11—C12	1.373 (2)
C1—H1	0.9300	C12—C13	1.445 (2)
C2—C3	1.370 (3)	C13—O1	1.2237 (19)
C2—Cl1	1.743 (2)	C13—O2	1.350 (2)
C3—C4	1.365 (4)	C14—O2	1.433 (2)
C3—H3	0.9300	C14—H14A	0.9600
C4—C5	1.385 (3)	C14—H14B	0.9600
C4—H4	0.9300	C14—H14C	0.9600
C5—C6	1.388 (2)	C15—N1	1.444 (2)
C5—H5	0.9300	C15—H15A	0.9600
C6—C7	1.525 (2)	C15—H15B	0.9600
C7—C12	1.511 (2)	C15—H15C	0.9600
C7—C8	1.542 (2)	C16—H16A	0.9600
C7—H7	0.9800	C16—H16B	0.9600
C8—C17	1.508 (2)	C16—H16C	0.9600
C8—C9	1.534 (2)	C17—O4	1.200 (2)
C8—H8	0.9800	C17—O5	1.319 (2)
C9—O3	1.430 (2)	C18—O5	1.451 (2)
C9—C10	1.514 (2)	C18—H18A	0.9600
C9—C16	1.525 (2)	C18—H18B	0.9600
C10—C11	1.507 (2)	C18—H18C	0.9600
C10—H10A	0.9700	N1—H1A	0.8600
C10—H10B	0.9700	O3—H4A	0.8200
			
C6—C1—C2	119.88 (16)	N1—C11—C12	123.34 (14)
C6—C1—H1	120.1	N1—C11—C10	115.45 (13)
C2—C1—H1	120.1	C12—C11—C10	121.20 (13)
C3—C2—C1	121.8 (2)	C11—C12—C13	119.52 (14)
C3—C2—Cl1	119.31 (16)	C11—C12—C7	122.84 (13)
C1—C2—Cl1	118.94 (16)	C13—C12—C7	117.55 (13)
C4—C3—C2	118.21 (19)	O1—C13—O2	120.94 (14)
C4—C3—H3	120.9	O1—C13—C12	127.01 (15)
C2—C3—H3	120.9	O2—C13—C12	112.03 (13)
C3—C4—C5	121.43 (19)	O2—C14—H14A	109.5
C3—C4—H4	119.3	O2—C14—H14B	109.5
C5—C4—H4	119.3	H14A—C14—H14B	109.5
C4—C5—C6	120.0 (2)	O2—C14—H14C	109.5
C4—C5—H5	120.0	H14A—C14—H14C	109.5
C6—C5—H5	120.0	H14B—C14—H14C	109.5
C1—C6—C5	118.74 (17)	N1—C15—H15A	109.5
C1—C6—C7	120.30 (13)	N1—C15—H15B	109.5
C5—C6—C7	120.86 (17)	H15A—C15—H15B	109.5
C12—C7—C6	113.42 (12)	N1—C15—H15C	109.5
C12—C7—C8	112.29 (12)	H15A—C15—H15C	109.5
C6—C7—C8	106.51 (12)	H15B—C15—H15C	109.5
C12—C7—H7	108.1	C9—C16—H16A	109.5
C6—C7—H7	108.1	C9—C16—H16B	109.5
C8—C7—H7	108.1	H16A—C16—H16B	109.5
C17—C8—C9	110.78 (12)	C9—C16—H16C	109.5
C17—C8—C7	109.55 (12)	H16A—C16—H16C	109.5
C9—C8—C7	111.98 (12)	H16B—C16—H16C	109.5
C17—C8—H8	108.1	O4—C17—O5	123.70 (16)
C9—C8—H8	108.1	O4—C17—C8	124.20 (15)
C7—C8—H8	108.1	O5—C17—C8	112.10 (14)
O3—C9—C10	105.67 (13)	O5—C18—H18A	109.5
O3—C9—C16	110.09 (13)	O5—C18—H18B	109.5
C10—C9—C16	110.33 (13)	H18A—C18—H18B	109.5
O3—C9—C8	111.62 (12)	O5—C18—H18C	109.5
C10—C9—C8	107.83 (12)	H18A—C18—H18C	109.5
C16—C9—C8	111.13 (14)	H18B—C18—H18C	109.5
C11—C10—C9	114.41 (13)	C11—N1—C15	126.10 (14)
C11—C10—H10A	108.7	C11—N1—H1A	117.0
C9—C10—H10A	108.7	C15—N1—H1A	117.0
C11—C10—H10B	108.7	C13—O2—C14	116.46 (14)
C9—C10—H10B	108.7	C9—O3—H4A	109.5
H10A—C10—H10B	107.6	C17—O5—C18	116.35 (16)
			
C6—C1—C2—C3	1.1 (3)	C8—C9—C10—C11	49.17 (18)
C6—C1—C2—Cl1	−178.36 (12)	C9—C10—C11—N1	161.63 (14)
C1—C2—C3—C4	−0.7 (3)	C9—C10—C11—C12	−17.9 (2)
Cl1—C2—C3—C4	178.76 (17)	N1—C11—C12—C13	−6.3 (2)
C2—C3—C4—C5	0.4 (3)	C10—C11—C12—C13	173.14 (14)
C3—C4—C5—C6	−0.6 (3)	N1—C11—C12—C7	177.30 (14)
C2—C1—C6—C5	−1.2 (2)	C10—C11—C12—C7	−3.2 (2)
C2—C1—C6—C7	175.25 (15)	C6—C7—C12—C11	−130.26 (16)
C4—C5—C6—C1	1.0 (3)	C8—C7—C12—C11	−9.5 (2)
C4—C5—C6—C7	−175.46 (17)	C6—C7—C12—C13	53.32 (18)
C1—C6—C7—C12	49.45 (19)	C8—C7—C12—C13	174.13 (13)
C5—C6—C7—C12	−134.17 (16)	C11—C12—C13—O1	13.8 (3)
C1—C6—C7—C8	−74.57 (17)	C7—C12—C13—O1	−169.67 (15)
C5—C6—C7—C8	101.81 (17)	C11—C12—C13—O2	−164.39 (14)
C12—C7—C8—C17	165.93 (13)	C7—C12—C13—O2	12.1 (2)
C6—C7—C8—C17	−69.36 (16)	C9—C8—C17—O4	62.5 (2)
C12—C7—C8—C9	42.61 (17)	C7—C8—C17—O4	−61.5 (2)
C6—C7—C8—C9	167.32 (12)	C9—C8—C17—O5	−116.62 (15)
C17—C8—C9—O3	−69.40 (16)	C7—C8—C17—O5	119.35 (15)
C7—C8—C9—O3	53.22 (16)	C12—C11—N1—C15	175.24 (17)
C17—C8—C9—C10	174.96 (12)	C10—C11—N1—C15	−4.3 (2)
C7—C8—C9—C10	−62.42 (16)	O1—C13—O2—C14	3.0 (2)
C17—C8—C9—C16	53.92 (17)	C12—C13—O2—C14	−178.66 (16)
C7—C8—C9—C16	176.54 (13)	O4—C17—O5—C18	−0.7 (3)
O3—C9—C10—C11	−70.31 (16)	C8—C17—O5—C18	178.47 (18)
C16—C9—C10—C11	170.72 (14)		

### Hydrogen-bond geometry (Å, °)

**Table:**

*D*—H···*A*	*D*—H	H···*A*	*D*···*A*	*D*—H···*A*
N1—H1A···O1	0.86	2.0	2.673 (2)	134
O3—H4A···O4	0.82	2.39	2.990 (2)	131
C15—H15B···O1^i^	0.96	2.52	3.327 (3)	142

Symmetry codes: (i) −*x*+1, −*y*+1, −*z*+1.
